# Predicting Factors for Seizures after Cerebral Venous Thrombosis: A Retrospective Single Center Cohort Study

**DOI:** 10.3390/life13010111

**Published:** 2022-12-30

**Authors:** Francesca Colò, Valerio Brunetti, Mariangela Di Muro, Elena Rossi, Francesca Bartolomei, Andrea Maria Alexandre, Simone Bellavia, Irene Scala, Artur Słomka, Fabio Pilato, Giovanni Frisullo, Aldobrando Broccolini, Giacomo Della Marca

**Affiliations:** 1 School of Medicine, Catholic University, 00168 Rome, Italy; 2 Department of Neurology, Fondazione Policlinico Universitario A. Gemelli IRCCS, 00168 Rome, Italy; 3 Department of Anesthesiology in Obstetrics, Gynecology, Pain Therapy, Fondazione Policlinico Universitario A. Gemelli IRCCS, 00168 Rome, Italy; 4 Department of Radiology, Oncologic Radiotherapy and Hematology, Fondazione Policlinico Universitario A. Gemelli IRCCS, 00168 Rome, Italy; 5 Neuroradiology Unit, Fondazione Policlinico Universitario A. Gemelli IRCCS, 00168 Rome, Italy; 6 Department of Pathophysiology, Nicolaus Copernicus University in Torun, Ludwik Rydygier Collegium Medicum, 87-100 Torun, Poland; 7 Unit of Neurology, Neurophysiology, Neurobiology, Department of Medicine, University Campus Bio-Medico, 00128 Rome, Italy

**Keywords:** cerebral venous thrombosis, hemorrhagic infarct, seizures, seizures recurrence

## Abstract

Background: Seizures are a common complication of cerebral venous thrombosis. In this study, we intended to define clinical and neuroradiological factors associated with early and late seizures and predictors for seizure recurrence. Methods: The database of our high-volume tertiary stroke center was screened for patients diagnosed with cerebral venous thrombosis between April 2006 and July 2021. Demographics, clinical, imaging, and instrumental data were collected. Results: Out of a total of 80 patients, 30 had seizures, either within the first week after onset (22 patients) or after (8 patients). Speech impairment and intracerebral bleeding were statistically associated with seizures in univariate analysis, but in a logistic regression model, only brain damage with hemorrhagic infarct and/or presence of brain hematoma [OR 6.051; 95% CI 1.881–19.468] (*p* = 0.003) were predicting factors for seizures. Late seizures were significantly more frequent in younger age [OR 0.864; 95% CI 0.763–0.978] (*p* = 0.020). Early seizures resulted as protective factors for recurrence; an altered state of consciousness at baseline and late seizures resulted as predictive factors for relapses (0.0% vs. 81.0%, *p* = 0.005, and 100.0% vs. 19.0%, *p* < 0.005, respectively). Conclusions: Our study confirms brain bleeding as the strongest risk factor for seizures after cerebral venous thrombosis. Recurrence is unusual after early seizures, while the presence of late seizures seems to raise the risk of recurrence.

## 1. Introduction

Cerebral venous thrombosis (CVT) is a rare cerebrovascular disorder that accounts for up to 3% of all strokes [1]. Nonetheless, its incidence may be underestimated because of the variability of symptoms and clinical course, which do not always lead to an appropriate neuroimaging study [2]. CVT usually involves young adults, and it is associated with several etiological conditions such as thrombophilia, pregnancy, puerperium, use of oral contraceptives, infections, and malignancy [3]. It affects more frequently the transverse, sigmoid, and sagittal sinuses as well as deep and superficial veins [1]. Venous congestion leads to brain infarction that is frequently characterized by signs of bleeding, either in the form of hemorrhagic transformation of an ischemic lesion or brain hematoma [2,3,4]. The prognosis is usually favorable, with a complete recovery from symptoms if anticoagulation is promptly started [3]. 

Seizures are a common complication of CVT, occurring in 34–50% of patients, either in the early phase of the disease or as a late symptom [5], with late seizures defined as events occurring after 7 days from the diagnosis [6,7]. Features associated with early seizures include evidence of hemorrhagic infarction on admission imaging, focal motor or sensory deficits, an altered state of consciousness, cortical vein thrombosis, focal edema/infarction, supratentorial lesions, and the involvement of the frontal lobe and superior sagittal sinus with high D-dimer levels [8,9,10]. On the other hand, predictors of late seizures are still elusive [11]. Defining predictors for late seizures, and in particular for seizure recurrence, is beneficial to plan treatment and long-term follow-up for these patients [6,7,8,9,10,11].

Hence, in this single-center retrospective study, we intend to define clinical and neuroradiological factors that can predict the occurrence of seizures during the acute phase of CVT and of long-term seizure recurrence.

## 2. Materials and Methods

### 2.1. Selection of Patients and Data Collection

This is a retrospective, single-center, observational cohort study that enrolled consecutive patients diagnosed with CVT in our high-volume tertiary stroke center between April 2006 and July 2021. All data were anonymized, and the work was conducted within the framework of a nonprofit study protocol approved by the ethics committee of the coordinator center (protocol number 6410/20, ID 3004). The need for informed consent was waived due to the retrospective nature of the study. The diagnosis was based on clinical symptoms and evidence of thrombosis in the cerebral venous system on computed tomography angiography (CTA) and/or magnetic resonance imaging (MRI). Demographics, time elapsed between onset of symptoms and diagnosis (lag time), baseline clinical, imaging, and instrumental data, as well as risk factors (including congenital or acquired thrombophilia) and type of anticoagulant therapy, were collected. Baseline clinical data included the presence and type of seizures, headache, motor deficit, altered consciousness, and speech impairment. Clinical outcome was evaluated by means of the modified Rankin Scale (mRS) at 90 days after the acute event, with an unfavorable outcome defined by a mRS score ≥ 1. CTA and MRI scans were reviewed to define the site and extent of venous thrombosis and the presence of parenchymal damage with or without hemorrhagic infarction in each patient. Venous Occlusion Image Score (VOIS), a quantitative scoring method for treatment guidance and outcome prediction [12], was calculated. We collected data about the presence of early or late seizures during the acute phase and relapses during the follow-up period. As regards patients with seizures, we considered the type and duration of the antiepileptic treatment, side effects, modification, or interruption of therapy.

### 2.2. Statistical Analysis

We described general characteristics of the population with summary statistics and used the Mann–Whitney U-test and the Pearson’s chi square test/Fisher’s exact test as appropriate to test differences between groups. We used the Shapiro–Wilk test as the normality test. We evaluated univariate associations between patients who experienced seizures (both early and late) and patients who did not, respectively. A multivariate analysis was performed with a logistic regression model using the condition of having seizures as a dependent variable. Despite age and sex, variables were chosen on a clinical basis and included the presence of brain hemorrhage, motor impairment, and speech impairment. The significance threshold was set at 5%. In order to determine the goodness of fit of the logistic regression model, the Hosmer–Lemeshow test was used. Furthermore, we tested univariate and multivariate associations for early and late seizures separately. As regards relapsing seizures, we evaluated, in univariate analysis, risk factors for recurrence in patients who presented both early and late seizures. Statistical analysis was performed using SPSS version 25 (IBM).

## 3. Results

Eighty patients (58 women, or 72.5%) diagnosed with CVT were enrolled in this study. All patients were treated with either i.v. sodium heparin or subcutaneous injection of low molecular weight heparin during the acute phase, according to standard protocols, and were eventually shifted to oral anticoagulants after discharge [13]. The time of follow-up went from 8 months for patients diagnosed in March 2021 to up to 13 years (a mean of 6.3 years; 65 patients were followed-up for more than 2 years). Eleven patients were lost to follow-up. Thirty patients (37.5% of the total) had seizures, either during the first week after diagnosis (22 patients, 73.4%) or later (8 patients, 26.7%). Demographics and risk factors were similar between the group of patients with seizures and that of patients without. Additionally, there was no significant difference in lag time. Clinical outcome was overall favorable in both groups, with a total of 47 patients (58.8%) having a 90 day mRS score of 0. Unfavorable outcomes (mRS ≥ 1) were associated with an altered state of consciousness, older age, and a lower VOIS score, indicating a more severe clot burden in univariate analysis.

In univariate analysis, the presence of speech impairment and intracerebral bleeding on admission imaging were associated with seizures in a statistically significant manner (50.0% vs. 26.0%, *p* = 0.029, and 80.0% vs. 42.0%, *p* < 0.001, respectively). In a logistic regression model, only brain damage with hemorrhagic infarction and/or presence of brain hematoma [OR 6.051; 95% CI 1.881–19.468] (*p* = 0.003) was associated with seizures (Table 1).

Regarding early seizures, no statistically significant association was found with baseline demographic and imaging features, whereas late seizures were significantly more frequent in younger age groups [OR 0.864; 95% CI 0.763–0.978] (*p* = 0.020). 

Twenty-eight patients were placed on anticonvulsant therapy (21 patients on levetiracetam, 6 on valproate, 1 on carbamazepine, and 1 on phenobarbital), with no relevant differences in terms of efficacy. Therapy was usually initiated during hospitalization, with the exception of one patient who was placed on anti-seizure medication during follow-up because seizures occurred during the follow-up observation. 

Seizure recurrence was present in four patients, all of whom had late seizures during the acute phase. Two of them were already under treatment, so that therapy was modified. Ongoing therapy was switched to other anti-seizure medications in two patients due to relapses or side effects. Because of relapsing seizure, the drug dose was increased in one patient. Dysgeusia and dizziness were separately reported as side effects in two patients treated with valproate. Therapy was discontinued in 12 patients after a seizure-free period of 2 years (mean 2.2 years). Of these, eight (66.7%) were among those with early seizures in the acute phase.

Early seizures resulted as protective factors for recurrence, while altered state of consciousness at baseline and late seizures resulted as predictive factors for relapses in univariate analysis (0.0% vs. 81.0%, *p* = 0.005, and 100.0% vs. 19.0%, *p* < 0.005, respectively) (Table 2). Unfortunately, we were not able to perform a multivariate analysis due to the limited number of patients with relapses. 

## 4. Discussion

Our study confirms that seizures are a common complication of the acute phase of CVT, being present in about one third of patients as an early (within 7 days from onset) or late symptom [14,15]. Nevertheless, the need for prophylactic treatment and/or long-term therapy in these patients has not been thoroughly addressed. The presence of cortical vein thrombosis, supratentorial lesions, intracerebral hemorrhage, motor or sensory deficits, severity of neurological involvement with an altered state of consciousness, younger age, hyperglycemia, or alcoholism have been associated with seizures [5,8,9,10,16]. Our study underlines that primarily hemorrhagic lesions, both in the form of hemorrhagic transformation of an ischemic lesion or a brain hematoma, are highly predictive of seizures during the acute phase. This confirms the epileptogenic role of blood extravasation, as already demonstrated in patients with hemorrhagic stroke or arterial ischemic stroke with hemorrhagic transformation, possibly due to the mechanical effects of the expanding hemorrhage and/or the irritation of the cortex caused by products of hemoglobin degradation. This may act in conjunction with other abnormalities occurring during acute ischemic damage that reduce seizure threshold, such as cellular ion dysfunction and increased glutamate [16,17]. 

The need to identify patients with CVT at risk for seizure recurrence is relevant, as a definition of specific predictors may determine whether or not epilepsy can be diagnosed and thus treated [11]. According to current guidelines, epilepsy should be diagnosed in the presence of one seizure and a >60% risk of relapse [18]. Our study points out that seizures occurring beyond 7 days from onset are highly predictive for relapses, as previously suggested [11]. Indeed, the presence of late seizures during the acute phase may be linked to structural and permanent modifications in neuro-excitability, in contrast to early seizures that are possibly related to metabolic changes associated with acute central nervous system (CNS) damage leading to cellular function instability [7,19]. Furthermore, our study confirms a younger age as a predictive factor, in particular for late seizures. Whether younger brains may have a greater epileptogenic potential needs to be elucidated [20]. Among our patients, 28 were placed on anticonvulsant therapy (usually valproate or levetiracetam) according to current evidence-based recommendations that suggest initiation of an anti-seizure medication when a single seizure, either early or late, occurs and a parenchymal lesion is present because of the high risk of recurrence [14].

Although there is still a lack of consensus over the choice among anti-seizure medications for patients with poststroke epilepsy, most of our patients were placed on levetiracetam without reporting side effects or relapses.

Where appropriate, antiepileptic treatment was discontinued after a seizure-free period of 2 years (mean 2.2 years), as recommended [21]. In particular, therapy was successfully discontinued in patients with early seizures; on the contrary, patients with late seizures specifically require long-term treatment.

Overall, we suggest that CVT patients with hemorrhagic supratentorial brain lesions should be strictly monitored in the acute phase to tackle possible epileptic complications. For example, if seizures are suspected or considered highly probable, long-term electroencephalographic monitoring should be considered. Furthermore, in case of late-onset seizures, long-term treatment seems reasonable due to an increased risk of recurrence. Indeed, although the presence of seizures appears to have no impact on the final outcome and long-term disability [8], the adoption of prophylactic measures based on the evaluation of specific predictors might improve the quality of life of patients.

The main limitation of our study is the relatively low number of patients (in accordance with the low frequency of the disease), leading to an underestimation of other factors involved in the onset and recurrence of seizures. Regarding the latter aspect (e.g., seizure recurrence), results might have been impaired by the loss of some patients during the follow-up period. Therefore, our study should be viewed as exploratory and hypothesis-generating and used with caution in real-world clinical practice. On the other hand, although this aspect has been previously explored, our study points out that primarily hemorrhagic lesions predict seizures. In addition, our cohort is peculiar in that all patients were followed-up on for several years in the same center, thus leading to the collection of homogeneous data concerning clinical and instrumental assessments, as well as treatment protocols.

## 5. Conclusions

Our study confirms brain bleeding as the strongest risk factor for seizures after CVT. Relapse is unusual in patients with early seizures, while the presence of late seizures seems to be a considerable risk factor for further recurrence that can possibly justify long-term treatment. However, further randomized trials of adequate size are needed to better define clinical and instrumental predictors of acute seizures in patients with CVT, thus helping physicians plan long-term personalized treatment.

## Figures and Tables

**Table 1:** Predicting factors for seizures, univariate and multivariate analysis.

	Patients with Seizures (Acute and Late)	Patients without Seizures (Acute and Late)	*p*-Value *
*Clinical and instrumental features*			
Number of patients	30	50	
*Demographics*			
Women, n/N (%)	20/30 (66.7%)	38/50 (76.0%)	0.365
Age, mean (±SD)	48.5 (±13.07)	48.5 (±13.07)	0.761
Lag in days, mean (±SD)	5 (±10.65)	5 (±10.65)	0.361
*Clinical and instrumental features*			
Headache, n/N (%)	24/30 (80.0%)	44/50 (88.0%)	0.332
Altered State of Consciousness, n/N (%)	14/30 (46.7%)	18/50 (36.0%)	0.346
Motor deficit, n/N (%)	19/30 (63.3%)	21/50 (42.0%)	0.065
Speech impairment, n/N (%)	15/30 (50.0%)	13/50 (26.0%)	**0.029**
*Risk factors*			
Oral contraceptives, n/N (%)	9/30 (30.0%)	15/50 (30.0%)	1.000
Pregnancy, n/N (%)	1/30 (3.3%)	0/50 (0.0%)	0.194
Puerperium, n/N (%)	2/30 (6.7%)	3/49 (6.1%)	0.905
Thrombophilia, n/N (%)	12/30 (40.0%)	27/50 (54.0%)	0.301
*Imaging data*			
Intracerebral hemorrhage or hemorrhagic transformation of infarcted brain tissue, n/N (%)	24/30 (80.0%)	21/50 (42.0%)	**<0.001**
Brain lesion w/o bleeding, n/N (%)	16/30 (53.3%)	19/50 (38.0%)	0.181
Deep venous systems thrombosis, n/N (%)	3/30 (10.0%)	8/50 (16.0%)	0.512
VOIS, median (IQR)	7 (5.0–7.3)	6 (5.0–7.3)	0.353
Follow-up			
Excellent outcome ^†^, n/N (%)	15/30 (50.0%)	32/50 (64.0%)	0.218
Death, n/N ^‡^ (%)	2/30 (6.7%)	1/50 (2.0%)	0.287
	**OR**	**95% CI**	***p*-Value**
*Multivariate logistic regression for predictors of seizures*			
Age	0.962	0.920–1.006	0.087
Sex	1.565	0.477–5.138	0.249
Intracerebral hemorrhage or hemorrhagic transformation of infarcted brain tissue	**6.051**	**1.881–19.468**	**0.003**
Motor deficit	1.493	0.485–4.594	0.485

Predicting factors for seizures after cerebral venous thrombosis: univariate and multivariate analysis. Data are presented as mean/median ± SD/IQR or as a percentage (%); SD, Standard Deviation; IQR, Interquartile Range; OR, Odds Ratio; CI, Confidence Interval; and VOIS, Venous Occlusion Image Score; * Statistical significance was considered at *p* < 0.05; ^†^ 3 month favorable outcome: modified Rankin Scale score = 0; ^‡^ 3 month mortality.

**Table 2:** Predicting factors for seizures recurrence, univariate analysis.

	Patients with Recurrence	Patients w/o Recurrence	*p*-Value *
*Clinical and instrumental features*			
Number of patients	4 ^*f*^	21 ^*f*^	
*Demographics*			
Women, n/N (%)	3/4 (75.0%)	14/21 (66.7%)	1.000
Age, mean (±SD)	49 (±13.07)	48 (±13.07)	0.363
Lag in days, mean (±SD)	3 (±10.65)	5 (±10.65)	0.628
*Clinical and instrumental features*			
Headache, n/N (%)	3/4 (75.0%)	18/21 (85.7%)	0.527
Altered State of Consciousness, n/N (%)	4/4 (100.0%)	8/21 (38.1%)	**0.039**
Motor deficit, n/N (%)	4/4 (100.0%)	12/21 (57.1%)	0.262
Speech impairment, n/N (%)	2/4 (50.0%)	12/21 (57.1%)	1.000
*Risk factors*			
Oral contraceptives, n/N (%)	2/4 (50.0%)	4/21 (19.0%)	0.234
Pregnancy, n/N (%)	0/4 (0.0%)	1/21 (4.8%)	1.000
Puerperium, n/N (%)	0/4 (0.0%)	2/21 (9.5%)	1.000
Thrombophilia, n/N (%)	1/4 (25.0%)	9/20 (45.0%)	0.614
*Imaging data*			
Intracerebral hemorrhage or hemorrhagic transformation of infarcted brain tissue, n/N (%)	4/4 (100.0%)	16/21 (76.2%)	0.550
Brain lesion w/o bleeding, n/N (%)	3/4 (75.0%)	10/21 (47.6%)	0.593
Deep venous systems thrombosis, n/N (%)	0/4 (0.0%)	3/21 (14.3%)	1.000
VOIS, median (IQR)	7 (5.0–7.3)	7 (5.0–7.3)	0.727
*Follow-up*			
Favorable outcome ^†^, n/N (%)	2/4 (50.0%)	12/21 (57.1%)	1.000
Death ^‡^, n/N (%)	0/4 (0.0%)	1/21 (4.8%)	1.000
*Seizures features during the acute phase*			
Early seizures, n/N (%)	0/4 (0.0%)	17/21 (81.0%)	**0.005**
Late seizures, n/N (%)	4/4 (100.0%)	4/21 (19.0%)	**0.005**
Generalized, n/N (%)	3/3 (100.0%)	15/20 (75.0%)	**1.000**

Predicting factors for seizure recurrence using univariate analysis. Data are presented as mean/median ± SD/IQR or as a percentage (%); SD, Standard Deviation; IQR, Interquartile Range; and VOIS, Venous Occlusion Image Score; * Statistical significance was considered at *p* < 0.05; ^†^ 3 month favorable outcome: modified Rankin Scale score = 0; ^‡^ 3 month mortality. ^*f*^ Of the 30 patients with seizures, 5 were lost to follow-up.

## Data Availability

The data that support the findings of this study are available from the corresponding author upon reasonable request.
